# Acetylation model predicts prognosis of patients and affects immune microenvironment infiltration in epithelial ovarian carcinoma

**DOI:** 10.1186/s13048-024-01449-6

**Published:** 2024-07-19

**Authors:** Xuan Wang, Xiaoning Li, Li Wei, Yankun Yu, Yeernaer Hazaisihan, Lin Tao, Wei Jia

**Affiliations:** First Affiliated Hospital, Department of Pathology and Key Laboratory for Xinjiang Endemic and Ethnic Diseases, Shihezi University, Shihezi University School of Medicine, Shihezi, China

**Keywords:** Epithelial ovarian carcinoma, Acetylation, Prognostic signature, Immune microenvironment, Immunotherapy

## Abstract

**Background:**

Epithelial ovarian carcinoma (EOC) is a prevalent gynaecological malignancy. The prognosis of patients with EOC is related to acetylation modifications and immune responses in the tumour microenvironment (TME). However, the relationships between acetylation-related genes, patient prognosis, and the tumour immune microenvironment (TIME) are not yet understood. Our research aims to investigate the link between acetylation and the tumour microenvironment, with the goal of identifying new biomarkers for estimating survival of patients with EOC.

**Methods:**

Using data downloaded from the tumour genome atlas (TCGA), genotypic tissue expression (GTEx), and gene expression master table (GEO), we comprehensively evaluated acetylation-related genes in 375 ovarian cancer specimens and identified molecular subtypes using unsupervised clustering. The prognosis, TIME, stem cell index and functional concentration analysis were compared among the three groups. A risk model based on differential expression of acetylation-related genes was established through minimum absolute contraction and selection operator (LASSO) regression analysis, and the predictive validity of this feature was validated using GEO data sets. A nomogram is used to predict a patient's likelihood of survival. In addition, different EOC risk groups were evaluated for timing, tumour immune dysfunction and exclusion (TIDE) score, stemness index, somatic mutation, and drug sensitivity.

**Results:**

We used the mRNA levels of the differentially expressed genes related to acetylation to classify them into three distinct clusters. Patients with increased immune cell infiltration and lower stemness scores in cluster 2 (C2) exhibited poorer prognosis. Immunity and tumourigenesis-related pathways were highly abundant in cluster 3 (C3). We developed a prognostic model for ten differentially expressed acetylation-related genes. Kaplan–Meier analysis demonstrated significantly worse overall survival (OS) in high-risk patients. Furthermore, the TIME, tumour immune dysfunction and exclusion (TIDE) score, stemness index, tumour mutation burden (TMB), immunotherapy response, and drug sensitivity all showed significant correlations with the risk scores.

**Conclusions:**

Our study demonstrated a complex regulatory mechanism of acetylation in EOC. The assessment of acetylation patterns could provide new therapeutic strategies for EOC immunotherapy to improve the prognosis of patients.

**Supplementary Information:**

The online version contains supplementary material available at 10.1186/s13048-024-01449-6.

## Background

Among gynaecological cancers, epithelial ovarian cancer (EOC) is the second most common cause of mortality [1, 2]. The 2020 edition of U.S. Cancer Statistics reported that there are more than 239,000 new annual cases of EOC (constituting 3.6% of all cancer cases) [3], leading to approximately 185,000 deaths (representing 4.3% of all cancer deaths) [1] worldwide. Because of ineffective screening methods and the absence of early recognizable clinical symptoms, the majority of patients with EOC are diagnosed at an early stage, with a 5-year survival rate of 29%. While platinum-based therapy serves as the initial treatment for EOC, approximately 70% of patients will relapse within 3 years [4].

Currently, molecular indicators such as CA125 [5], HE4 [6], and BRCA1 are used for clinical monitoring. However, these biomarkers are not presently utilized as therapeutic targets. Moreover, single-gene predictive models often exhibit low specificity, whereas multigene-based prediction models demonstrate improved accuracy. With increasing availability of next-generation sequencing (NGS) data, multigene-based prediction models are becoming more specific than single-gene prediction models, offering enhanced predictive accuracy. Recently, several studies have identified the potential of utilizing multi-target combination therapy to enhance the prognosis of patients with tumours. Therefore, it remains crucial to explore new potential anti-tumour targets.

Many epigenetic studies have shown that the development and prognosis of EOC are influenced by the dynamic regulation of various oncogenes and tumour suppressor genes [7]. Epigenetic modification refers to the heritable phenotype that influences gene expression without modifying the DNA sequence. This regulation of tumour-related genes makes them possible therapeutic targets [8].

Acetylation modification is an invertible homeostasis process regulated by histone acetyltransferases (HATs) and histone deacetylases (HDACs) [9]; HATs facilitate the transfer of acetyl groups onto the N-terminal lysine residues, thereby counteracting the positive charge on these residues and unfolding the DNA conformation. This process loosens the structure of nucleosomes and activates the transcription of specific genes, making transcription factors more easily bind to promoter regions, while HDACs act in the opposite direction[10]. Abnormal expression of HATs and HDACs is related to malignant progression of tumours [11]. It has been identified as a key target for tumourigenesis and represents a novel class of anti-tumour drugs with wide-ranging applications [12]. In addition, a regulatory imbalance between HATs and HDACs is associated with EOC pathogenesis [13, 14]. Many studies have found that histone deacetylase inhibitors (HDACis), such as trichostatin A [15] and belinoist [16], act as important epigenetic regulatory drugs in cell proliferation, differentiation, cell cycle, and immune response, demonstrating significant anticancer potential. At present, there are three HDACis available for treating EOC [17]. Acetylation homeostasis is closely associated with the pathogenesis of EOC. However, there is a lack of systematic and holistic research investigating the impact of acetylation-related genes on EOC prognosis. Therefore, it is crucial to develop highly selective targeted drugs for EOC treatment.

Immunotherapy targeting programmed cell death protein 1(PD-1) and its ligand programmed cell death protein 1(PD-L1) are expected to improve the long-term survival prospects of patients with EOC in the future [18]. Many immune checkpoint inhibitors (ICI) are applied to treat tumours. However, owing to the specificity of immunotherapy drugs, only a minority of patients experience positive outcomes from these treatments. Furthermore, some tumours, such as ovarian, breast, and pancreatic cancers, appear to be inherently resistant to ICI drugs [19]. Approximately 20% of patients exhibit an objective response to immune checkpoint blockade (ICB). Recently, targeted therapy combined with immunotherapy has emerged as a crucial treatment for many advanced cancers owing to its advantages such as high targeting and low toxicity [20].

In our study, a new prognostic marker for acetylation-related differential gene regulation was established using samples from TCGA and GTEx databases. By combining expression profiles with prognostic information, acetylation-related genes associated with the prognosis of EOC were identified. Patients with EOC were divided into two groups, those with high risk and those with low risk. Genetic characteristics were determined through the application of LASSO Cox regression analysis. To predict the outcome of EOC, a prognostic model based on risk score was constructed. Using the CiberSort, Estimate, and ssGSEA algorithms, we assessed the differences in the immune and survival traits among distinct risk subgroups in tumour immune evasion. The study also examined the relationships between drug sensitivity, immune microenvironment, and EOC prognosis. Our study provides new prognostic markers for patients with EOC to lead to more effective treatment.

## Methods

### Data sources

Relevant collections of reactome genes were searched in the Human Genome Database (GeneCards, https://www.genecards.org/) using the search term “ACETYLATION”. In total, 4056 acetylation-related genes (ARGs) were downloaded from the GeneCards database [21]. Transcriptome sequencing data and relevant clinical data for 379 ovarian cancer patients were acquired from the Cancer Genome Atlas database (https://portal.gdc.cancer.gov/). In the absence of data from the normal group, we used the GTEx database to procure sequence data from the ovarian tissues of 88 unaffected female patients (accessed from https://xenabrowser.net). Normalisation of expression matrices for the independent datasets were carried out using the “SVA” package. Transcriptional data and associated clinical data for the immunotherapy cohort IMvigor210 were acquired from the R package “IMvigor210 CoreBiologies” [22, 23]. The Validation of immunohistochemical results for genes with prognostic value was performed by using the Human Protein Atlas (http://www.proteinatlas.org/).

### Screening of DEGs and cluster analysis

The R package “LIMMA” facilitated the screening of 3559 differentially expressed genes (DEGs), defined by |log2 fold change (FC)|> 1.5 and false discovery rate (FDR) < 0.01.Prognostic significance of DEGs was evaluated through univariate cox regression analysis. Unsupervised consensus clustering was performed using the R package “ConsensusClusterPlus”.

### Enrichment analysis

GSEA software (version 4.2.3) was used to study the differences in activated signalling pathways, utilizing hallmark gene sets and c2kegg gene sets. The annotated gene sets were extracted from the Molecular Signature Database (https://www.gsea-msigdb.org/gsea). The enriched functions and pathways were compared among the three clusters, with the screening criteria set at (NES)|> 1, *p* < 0.05, and FDR < 0.25. A comprehensive workflow diagram is shown in Supplementary Fig. 1.

### Immune pattern analysis

The single sample Genomic Enrichment Analysis (ssGSEA) software package “Genomic Variation Analysis (GSVA)” was used to calculate the relative enrichment of different immune cell types. The genes that serve as markers for various immune cells and their respective roles are described in Table S1 [24, 25]. Stromal and immune cells in the tumour tissue were estimated using expression data (ESTIMATE) to compute the tumour stroma score, immune score, and tumour purity. Cell type identification by estimating relative subsets of RNA transcripts (CIBERSORT) was employed to investigate immunological features. The immune characteristics among clusters 1 (C1), C2, and C3 were analysed by using the “IOBR” package [26]. The CIBERSORT source file (https://cibersort.stanford.edu/) was downloaded and processed using the R software. The full gene expression matrices from GTEx and TCGA datasets were used to estimate the relative proportions of the 22 immune cells in two distinct risk groups. The R package “ESTIMATE” was performed to predict the immune scores and tumour purity of the sample.

### Construction and validation of acetylation-related gene signatures

In the TCGA cohort, gene expression data of each patient with corresponding overall survival time and survival state information were combined. A univariate cox regression model was used to identify the prognostic genes, and genes with *p* < 0.1 were considered as prognostic markers through the LASSO cox regression model provided by the “glmnet” R package. The risk score for genetic traits was calculated using the formula:

$$\mathrm{Risk}\;\mathrm{Score}={\textstyle\sum_{\mathrm i}^{\mathrm n}}\,\mathrm{Coenfficient}\;\left(\mathrm{mRNAi}\right)\times\mathrm{Expression}\;\left(\mathrm{mRNAi}\right)$$

The patients were clustered into high-risk and low-risk groups according to the median risk score, excluding five patients without corresponding survival information. We verified the accuracy of the model by comparing the likelihood of survival in two different risk groups through Kaplan–Meier analysis. Receiver operating characteristic (ROC) curves were designed using the “survival” and “Time ROC” R software packages to assess the sensitivity and specificity of risk scores.

### Independent prognostic analysis

Univariate and multivariate cox regression models, implemented through the “survival” R software package, were used to estimate the independent prognostic value of risk scores. Collinearity analysis was performed using an interactive gene expression profiling analysis (http://gepia.cancer-pku.cn). The Nomogram generated utilizing the “survival” and “rms” packages illustrated the results of the predictive model, and calibration curves were drawn to determine the agreement between anticipated and observed prediction outcomes.

### Stemness index analysis

The RNA stemness index (mRNAsi) was computed using a one-class logistic regression machine-learning (OCLR) algorithm. The epigenetic regulatory mRNA stemness index (EREG mRNAsi), developed by Malta et al. [27], was employed, and Spearman’s correlation (RNA expression data) was used for statistical analysis. The stemness index was mapped to a range based on the TCGA database by subtracting minimum values and separating the results by maximum value. RNA-seq data for pluripotent stem cell samples were obtained from the Progenitor Cell Biology Consortium (PCBC) database [28].

### Immunotherapy response evaluation

As multiple immune checkpoint inhibitors can enhance anti-tumour immune activity, we employed the TIDE (http://tide.dfci.harvard.edu/) algorithm for assessing the potential clinical efficacy of immunotherapies [29, 30]. The IMvigor210 was used as an external validation set to validate the effects of immunotherapy and the reliability of predictive outcomes [31].

### Analysis of drug sensitivity

We utilized the R software package “oncopredict” for assessing chemotherapeutic responses, determining the half-maximal inhibitory concentration (IC_50_) for each patient by using the Cancer Drug Sensitivity Genomics website (GDSC, https://www.cancerrxgene.org/) [32–34].

#### Mutation analysis

We extracted mutation profiles of EOC samples from the TCGA database. The “mafTools” software package in the MAF format aided in visualising the mutation frequency within the high- and low-risk groups. Subsequently, we examined the relationship between the risk score and TMB. We also performed a KM survival analysis to compare the variations in OS among various groups based on TMB and risk scores [29].

#### Statistical analysis

The Wilcoxon test was used to compare gene expression levels between the two groups. The Chi-square test examined the correlations between acetylation-related DEGs (ARDEGs) and clinical parameters as well as the relationship between immunotherapy efficacy and risk scores. Additionally, KM survival analysis was used to assess the OS between different risk groups. Calibration, C-index, and ROC curves were employed to assess the predictive reliability of the risk and nomogram models. Univariate and multivariate cox regression models allowed for identification of ARDEGs and assessment of the independent prognostic value of the risk model. All statistical analyses were conducted using R software (version 4.0.2). The statistical significance was established at a level of *p < 0.05. The significance levels were defined as **p < 0.001; **p < 0.01.

## Results

### Differential expression analysis of ARGs in EOC

**Fig. 1:**
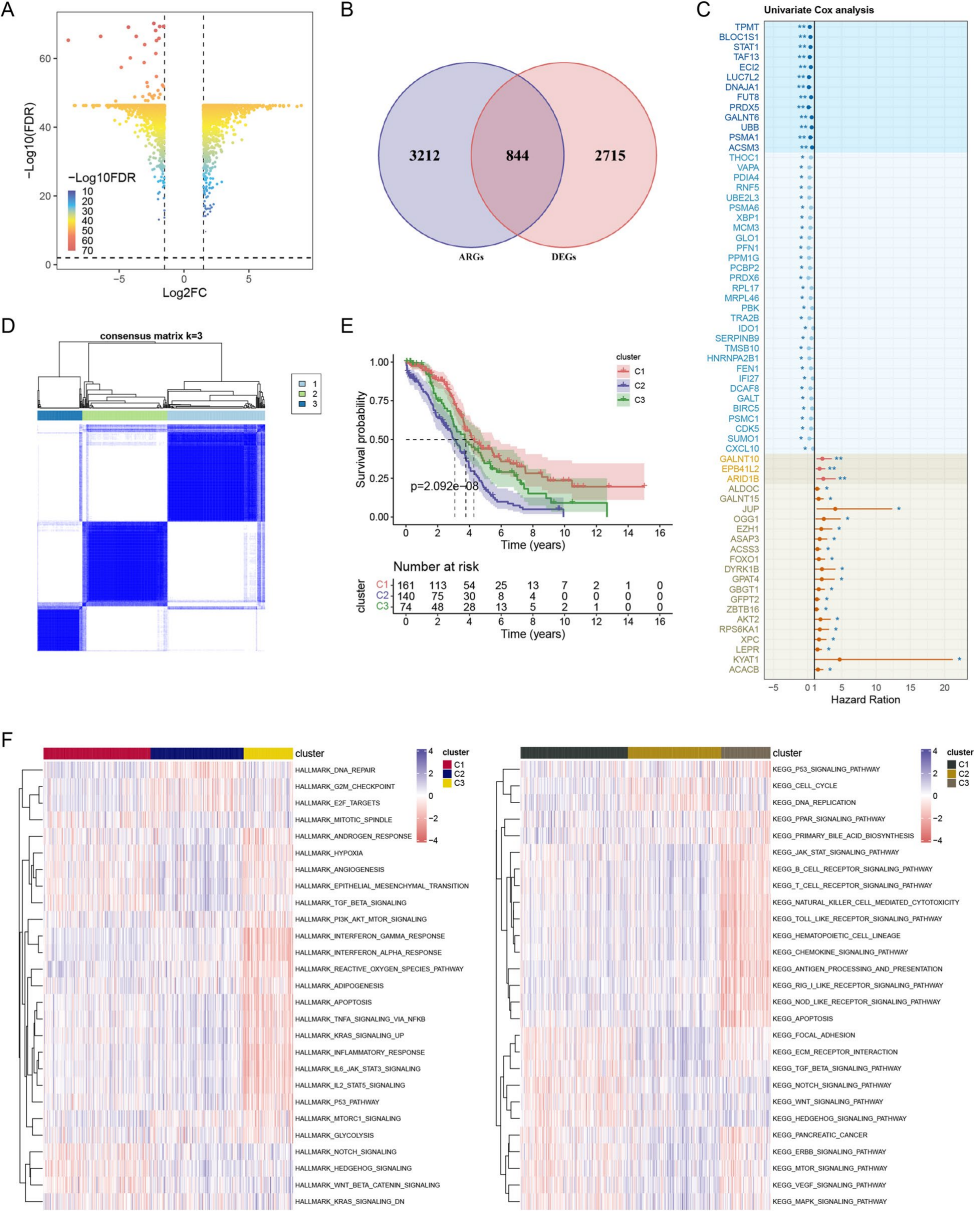
Differential expression analysis of ARGs in EOC. **A** Volcano map of DEGs. **B** The number of acetylation-related and differentially expressed genes is shown in a Venn diagram. **C** Prognostic values of 65 signatures are shown as forest plots of HR by univariate cox regression analysis. **D** Unsupervised clustering of 844 ARDEGs with prognostic value and consensus matrix when k = 3. **E** Overall survival rates among the three clusters are depicted by Kaplan–Meier analysis. **F** Bio-enriched pathway status in the three clusters is depicted by GSEA**.** The pathways that have been activated are denoted in red, while the pathways that have been inhibited are depicted in blue. (The notation “ns” signifies no significance; *p < 0.05; **p < 0.01; *** *p* < 0.001; ****p < 0.0001)

We acquired 844 ARDEGs were acquired from the intersection of 4056 ARGs and 3559 DEGs. Among 3559 DEGs, 1722 genes exhibited higher expression levels, while the other 1835 were downregulated (Fig. 1A, B). The prognostic significance of ARDEGs was analysed using a univariate Cox regression model. We identified 65 genes that were significantly associated with survival prognosis (*p* < 0.1) (Fig. 1C).

Unsupervised clustering was performed by determining k = 3 as the optimal value. Cophenetic correlation coefficients were subsequently calculated, categorising patients with EOC into three clusters: C1, C2, and C3 (Fig. 1D). To determine the latent biological behavioural differences underlying the different acetylation patterns, we used GSEA analysis to reveal pathways and functions enriched for these genes. C1 and C2 were abundant in pathways associated with immunity, including B cell receptors, T cell receptors, natural killer cell-mediated cytotoxicity, and apoptosis. Conversely, C3 exhibited an increase in pathways associated with tumourigenesis and cell proliferation, such as hedgehog signalling and DNA replication. The significantly enriched pathways in Clusters 1 and 2 included adaptive immune response, NF-κB signalling pathway, and P53 signalling pathway (Fig. 1F). Survival analysis indicated that patients in C1 had a more favourable prognosis than those in the other two clusters (log-rank *P* = 0.000000021, Fig. 1E), suggesting that the clusters impact the progression of EOC by affecting immune infiltration and stemness maintenance.

### Features of acetylation in immunotherapy and chemotherapy

**Fig. 2:**
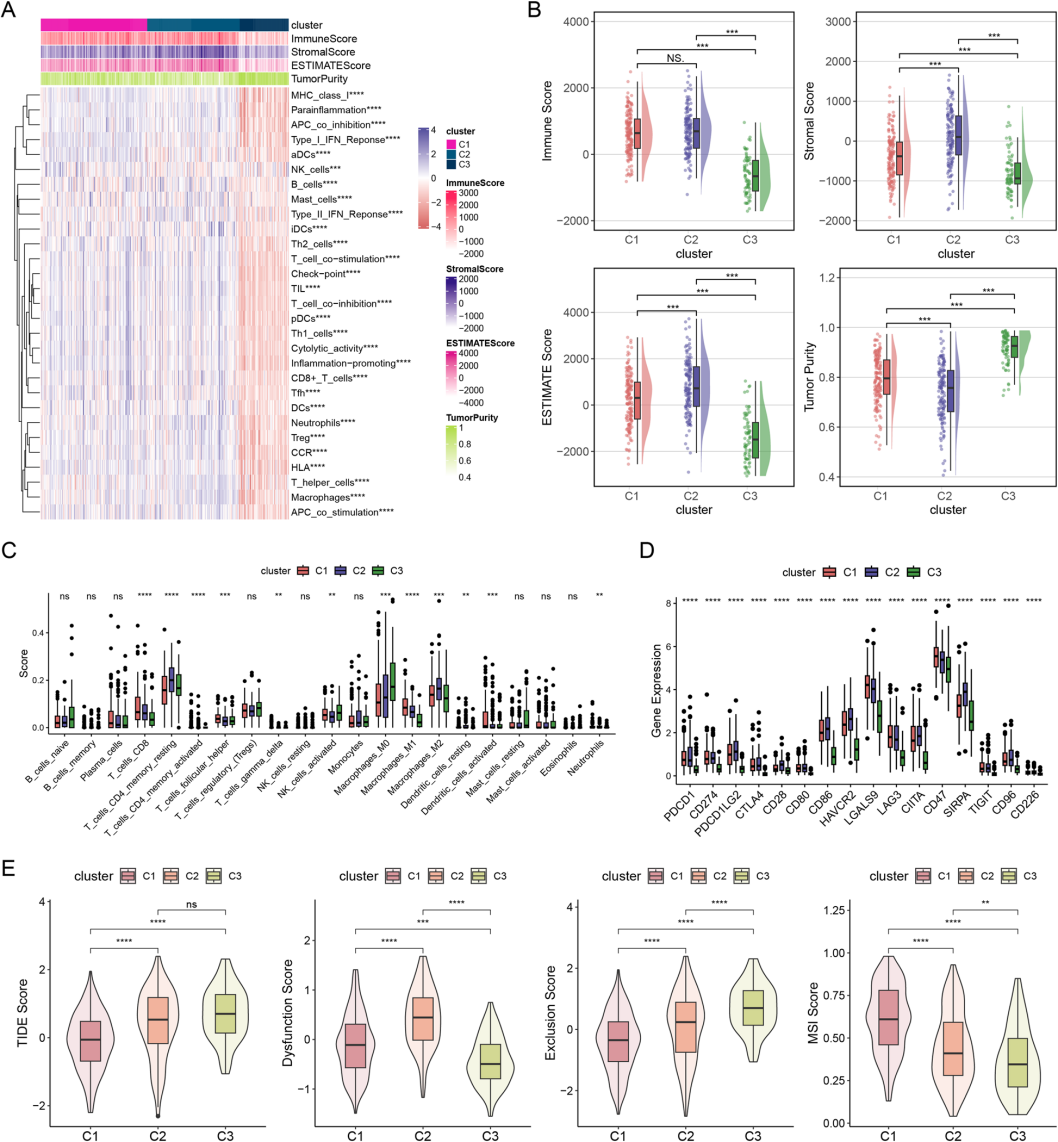
Features of acetylation in immunotherapy and chemotherapy. **A** The relative levels of immune cell infiltration in the three clusters using ssGSEA. **B** The ESTIMATE algorithm was employed to compare tumour immunity scores. **C** Immune cell components of different subtypes were determined using the CIBERSORT algorithm. **D** The levels of gene expression related to the immunotherapy target. **E** Differences in TIDE scores and microsatellite instability (MSI) scores

The ovarian TME landscape was systematically constructed and evaluated using the ssGSEA algorithm, which relied on 29 immune-related cell markers for analysis [35] (Fig. 2A, Supplemental Table 1). C1 and C2 exhibited notably elevated immune, stromal, and ESTIMATE scores, whereas the tumour purity of C3 was strikingly higher than that of the other clusters (Fig. 2B). C2 exhibited an immunosuppressive subtype characterised by an abundance of quiescent memory CD4 + T cells and M2 macrophages, along with elevated immune and stromal scores. However, C1 exhibited increased levels of anti-tumour components in TME, including CD8 + T cells, activated memory CD4 + T cells, T follicular helper cells, M1 macrophages, and activated dendritic cells. C3 exhibited moderate TME infiltrations such as M0 macrophages and stromal scores (Fig. 2C). TAMs and Tregs are prominent immunosuppressive cells in TME, known to contribute to tumorigenesis and immune evasion [36, 37]. Given that C2 and C3 were significantly enriched in TAMs and Tregs, we hypothesised that these two immunosuppressive cell types may significantly contribute to the poor clinical prognosis observed in patients exhibiting this acetylation model. Moreover, immune checkpoints were increased in C2 compared to C1 and C3, including PDCD1, CD274, PDCD1LG2, CTLA4, CD28, CD80, CD86, HAVCR2, LGALS9, LAG3, CIITA, CD47, SIRPA, TIGIT, CD96, and CD226 (Fig. 2D). The potential clinical efficacy of immunotherapy was assessed using the TIDE algorithm. Patients in C2 and C3 had higher TIDE scores and responded better to ICB immunotherapy (Fig. 2E). In conformity with our earlier findings, patients in C2 and C3 did not benefit from immunotherapy, validating the higher overall benefit in C1. These results reveal that ARDEGs may serve as potential predictors of the response to immunotherapy.

**Fig. 3:**
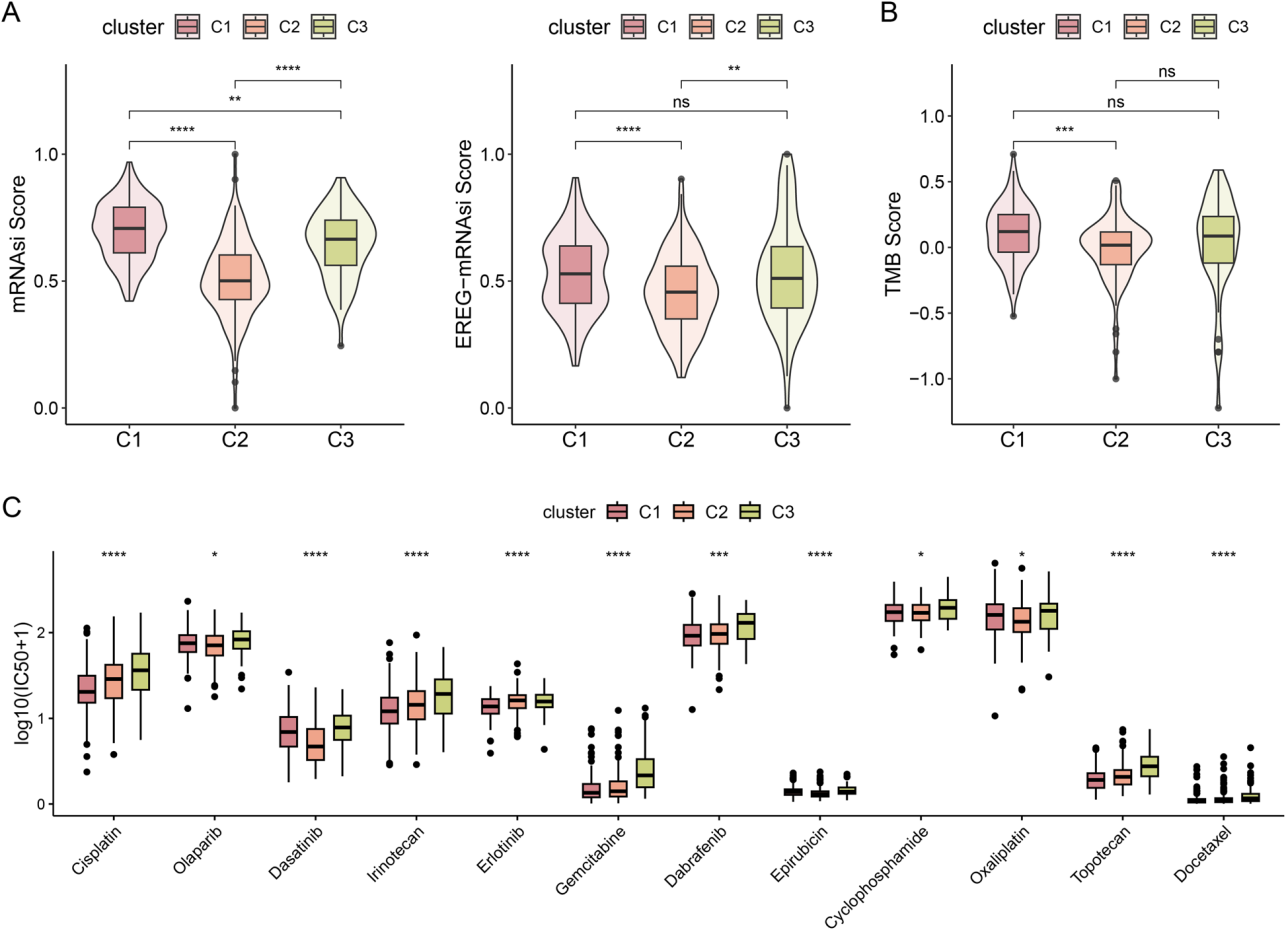
Development of prognostic gene signature. **A** Different stemness scores for C1, C2, and C3. **B** TMB differences between the three clusters. **C** Differences in the IC_50_ values of chemotherapeutic agents for the three clusters

Stemness analysis indicated that the stemness level was higher in C1 than in C2 and C3. We also investigated another stemness index, the EREG mRNAsi score, which indicated the same result (Fig. 3A). The TMB is an essential indicator used to assess clinical immunotherapy. The TMB was significantly increased in C1 (Fig. 3B). We examined the correlation between the pattern of acetylation and the response to chemotherapy. Several commonly used chemotherapeutic drugs, including doxorubicin, mitomycin C, and gemcitabine [38], and adjuvant treatments after surgery for EOC, such as cisplatin, methotrexate, and vinblastine [39], have been confirmed. We found that C3 was related to a high IC_50_ for cisplatin, dasatinib, irinotecan, gemcitabine, epirubicin, topotecan, docetaxel, and dabrafenib, indicating higher sensitivity to chemotherapy, but erlotinib exhibited the opposite trend (Fig. 3C). These results indicate a distinctive role of the acetylation pattern in predicting the efficacy of immunotherapy and chemotherapy, which could have direct application in clinical treatment.

### Development of prognostic gene signature

**Fig. 4:**
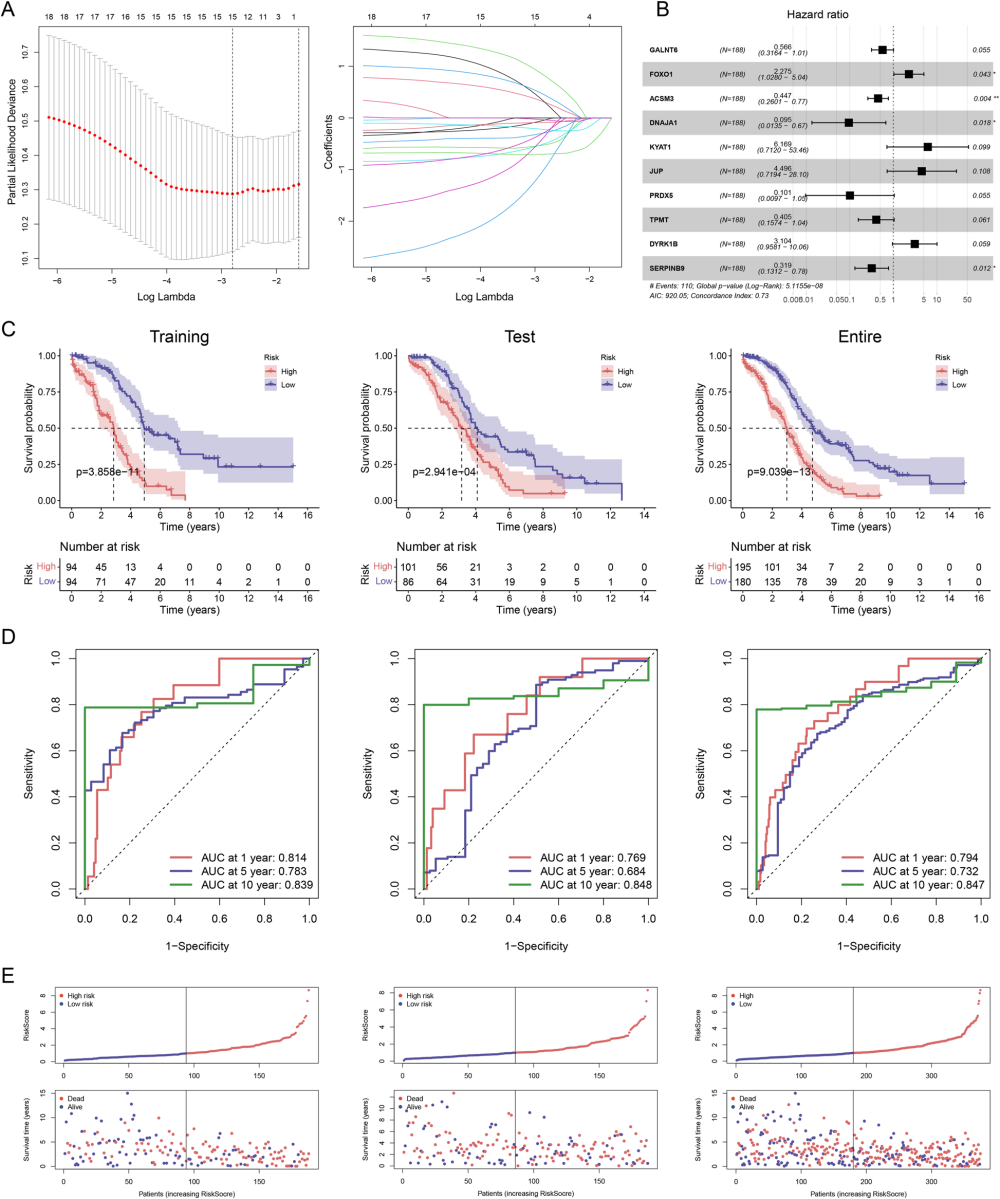
Independent prognostic role of model and model-based construction of nomograms. **A** Prognostic signature construction using LASSO cox regression analysis in a TCGA dataset. The wavelength is expressed in horizontal coordinates, and the coefficient is expressed in vertical coordinates. **B** Multivariate cox regression analysis of the ARDEGs. **C**,** D**,** E** Differential outcomes in risk score, survival time, and survival state for the training, testing, and entire set. Kaplan–Meier analysis ©. ROC curves of the risk signature. The AUC for 1, 5, and 10 years was obtained, which indicated a better discrimination ability

The LASSO Cox regression model was applied to establish the risk features (Fig. 4A). Genes contributing less to the model were removed based on the AIC criteria. As a result, 10 genes were obtained based on the optimum λ value: *GALNT6A*, *FOXO1*, *ACSM3*, *DNAJA1*, KYAT1, *JUP*, *PRDX5*, *TPMT*, *DYRK1B*, and *SERPINB9* (Fig. 4B). Using the coefficient for each gene, the risk score was calculated using the following equation:

$$\mathbf{Risk}\boldsymbol\;\mathbf{score}=(-0.5693)\ast GALNT6A(\exp)+(0.8221)\ast FOXO1(\exp)+(-0.8049)\ast ACSM3(\exp)+(-2.3505)\ast DNAJA1(\exp)+(1.8196)\ast KYAT1(\exp)+(1.5032)\ast JUP(\exp)+(-2.2938)\ast PRDX5(\exp)+(-0.9027)\ast TPMT(\exp)+(1.1327)\ast DYRK1B(\exp)+(-1.142)\ast SERPINB9(\exp).$$

According to the median risk score, patients with EOC were separated into high-risk and low-risk groups. The survival analysis showed a notably lower OS rate in the high-risk group (*p* < 0.001; Fig. 4C). Similarly, patients with high risk exhibited lower survival rates and shorter survival times than those with low risk (Fig. 4E). Subsequently, ROC curves were performed (Fig. 4D) to determine the prophetic accuracy of the risk score. The areas under the curve (AUC) values for the signatures at 1, 5, and 10 years were 0.794, 0.732, and 0.847, respectively.

### Independent prognostic role of model and model-based construction of nomograms

**Fig. 5:**
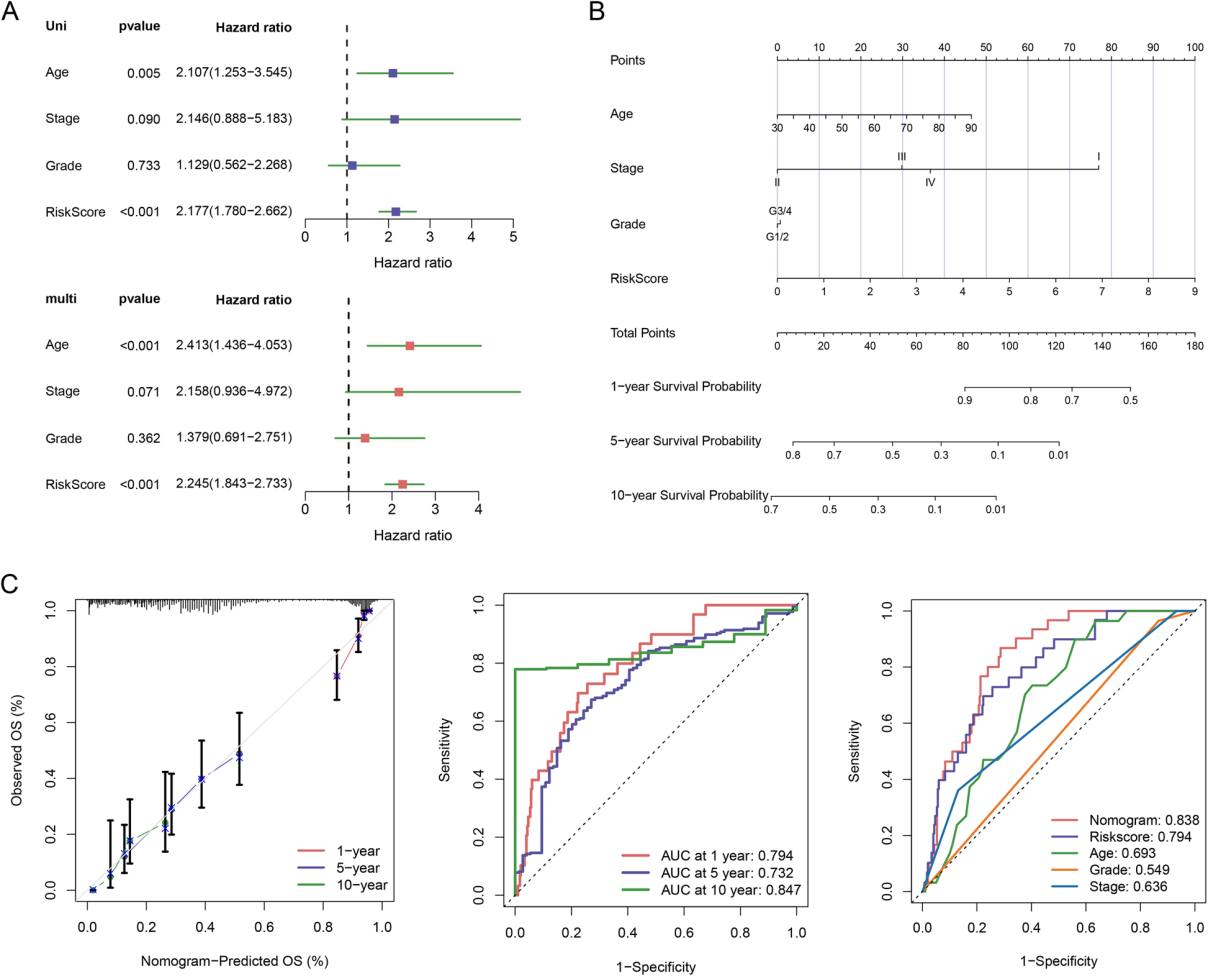
Patterns of immune cell infiltration in patients with EOC. **A** Prognostic values of ten signatures shown as forest diagrams of hazard ratios by univariate and multivariate cox regression analyses of EOC. **B** Predicting the survival outcomes of patients at 1, 5, and 10 years based on nomograms of age, stage, grade, and risk scores. Adding these four points to the total points could predict the survival probability. **C** Calibration curves of the nomograms drawn based on the agreement between 1-, 5-, and 10 years of predictions and observations. ROC curves were used to forecast the survival prognosis of patients at 1-, 5- and 10 years

Clinical features such as age and tumour stage were included in regression models to assess the independent association of the risk scores with OS. Univariate analyses suggested that age and risk score were related to prognosis. In the multivariate cox regression model, these two factors emerged as independent indictors of prognosis (HR for risk score, 2.245; 95% CI, 1.843–2.733; *p* < 0.001; Fig. 5A). Genetic correlation is illustrated in Supplementary Fig. 3, showing that all genes were independent of each other, exhibiting low covariance and thus being eligible for inclusion in the model.

Based on the above findings, nomogram features and clinical characteristics (age, stage, tumour grade, and risk score) were established to forecast survival rates at 1, 5, and 10 years (Fig. 5B). Calibration plots of this model performed satisfactorily, with C-indices at 1, 5, and 10 years were 0.794, 0.732, and 0.847, respectively, demonstrating superior predictive value. AUC for the nomogram model, age, stage, and grade were 0.838, 0.693, 0.549, and 0.636, respectively. Comparison of nomograms, risk scores, and clinical parameters indicated that the nomogram and risk scores were more effective in forecasting the long-term prognosis of patients with EOC (Fig. 5C).

### Patterns of immune cell infiltration in patients with EOC

**Fig. 6:**
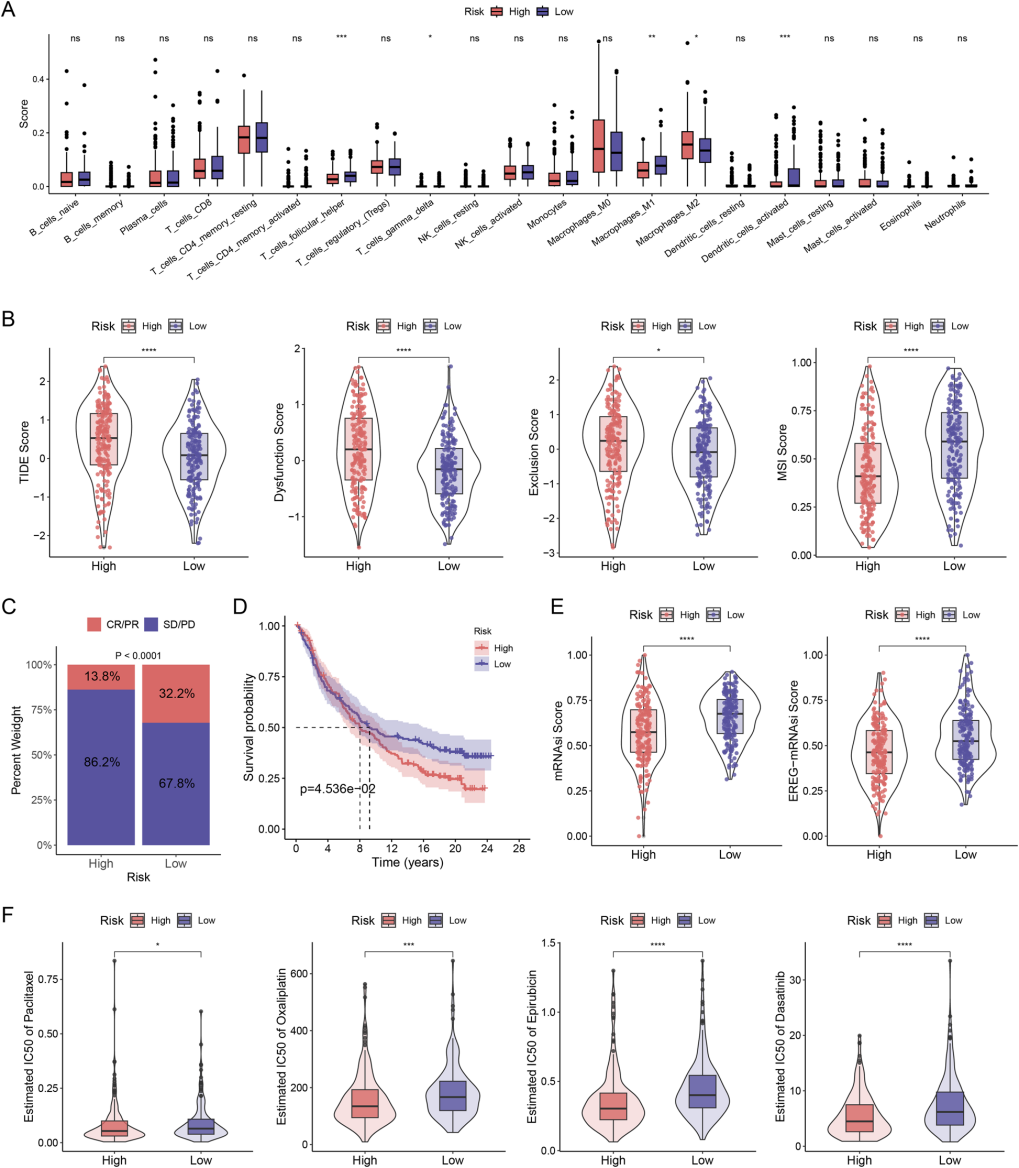
Mutational characterization and risk groups for ARDEGs. **A** Enrichment of TME-infiltrating cells in two risk groups. **B** Differential outcomes of TIDE scores and MSI scores. **C** Differences in response to the four immunotherapies in the IMvigor 210 cohort (using the Kruskal–Wallis H test). **D** KM survival indicated that the two distinct risk groups were notably related to OS. **E** Different scores of tumour stemness index. **F** Sensitivity to differential chemotherapeutic drugs

We explored differences in the infiltrations of 21 immune cells between patients with high and low risk using the CIBERSORT algorithm. Multiple types of immune cells, including follicular helper T cells, activated dendritic cells, M1 macrophages, and gamma-delta T cells, exhibited remarkably higher infiltration densities in the low-risk group (Fig. 6A). This indicates heightened sensitivity to immunotherapy in the low-risk group, whereas the high-risk group may exhibit reduced sensitivity owing to M2 macrophage-mediated immunosuppressive effects. The identified risk signature correlated with immune cell infiltration, which is essential for predicting immunotherapy responses. We explored its relation to anti-PD-L1 immunotherapy response, revealing a lower survival rate in the high-risk group, indicating unsuitability for immunotherapy (Fig. 6B). In the IMvigor210 cohort, patients classified as having low risk demonstrated notable therapeutic outcomes and favourable clinical responses to immunotherapy (Fig. 6C, D). These results propose that risk scores could serve as indicators of immunotherapy response. Nevertheless, there was no obvious difference in survival rate between the two groups, which may be due to an insufficient sample size (Fig. 6E). We further examined the variation in 24 chemotherapeutic and targeted drugs, indicating a striking difference in estimated IC_50_ values between the high-risk and low-risk groups. The high-risk group exhibited lower IC_50_ values for paclitaxel, oxaliplatin, epirubicin, and dasatinib (Fig. 6F), which implied a higher sensitivity to chemotherapy. These results may guide the selection of immunotherapy and chemotherapy for patients with EOC.

### Mutational characterization and risk groups for ARDEGs

**Fig. 7:**
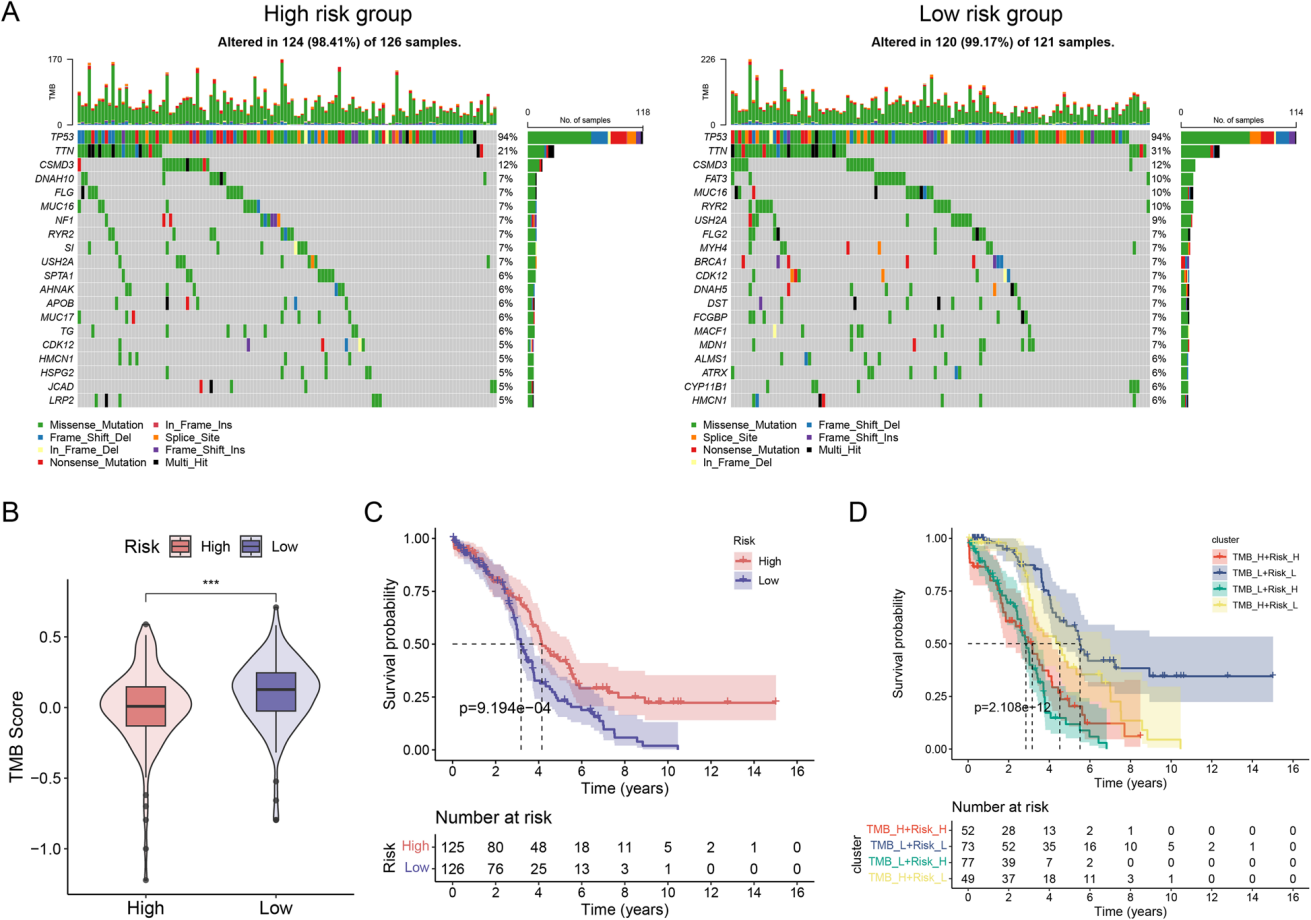
Validation of Prognostic Genes Using Clinical Tissue Samples. **A** The differential distribution of tumour somatic mutations is shown. Each column represents one patient. The top bar chart shows the TMB. The right figures and bar chart show the mutation rates and the proportions of change, respectively, for each gene. **B** Differences in TMB scores. **C** Survival analyses by KM survival for two distinct risk groups. **D** Survival analysis of four groups based on tumour mutation load and risk score

Gene mutations are major contributors to tumourigenesis and tumour progression. We conducted an analysis to compare the distribution of somatic mutations in the TCGA-OV cohort between high- and low-risk groups by using the maftools R package. Evaluating the frequency of tumour mutations, we computed the TMB scores for patients. Based on the established model, the proportions of somatic mutations in the high-risk group were TP53 > TTN > CSMD3 > DNAH10 > FLG > MUC16 (CA125) > NF1 > RYR2 > SI > USH2A (Fig. 7A), whereas those in the low-risk group were TP53 > TTN > CSMD3 > FAT3 > MUC16 (CA125) > RYR2 > USH2A > FLG2 > MYH4 > BRCA1 (Fig. 7B). We observed that TMB, a crucial metric that is used in current clinical practice for evaluating immunotherapy, was remarkably higher in the low-risk group compared to that in the high-risk group. Furthermore, TMB was found to be correlated with the survival of patients (Fig. 7C, D).

The combination of TMB and risk scores was a robust predictor of clinical outcomes in patients with EOC (Fig. 7D). Patients with a high TMB and low risk scores exhibited more favourable survival rates, whereas those with a low TMB and high risk scores demonstrated worse survival rates. These results suggest that risk scores and TMB are useful in the prediction of prognosis for EOC patients.

### Validation of prognostic genes using clinical tissue samples

**Fig. 8:**
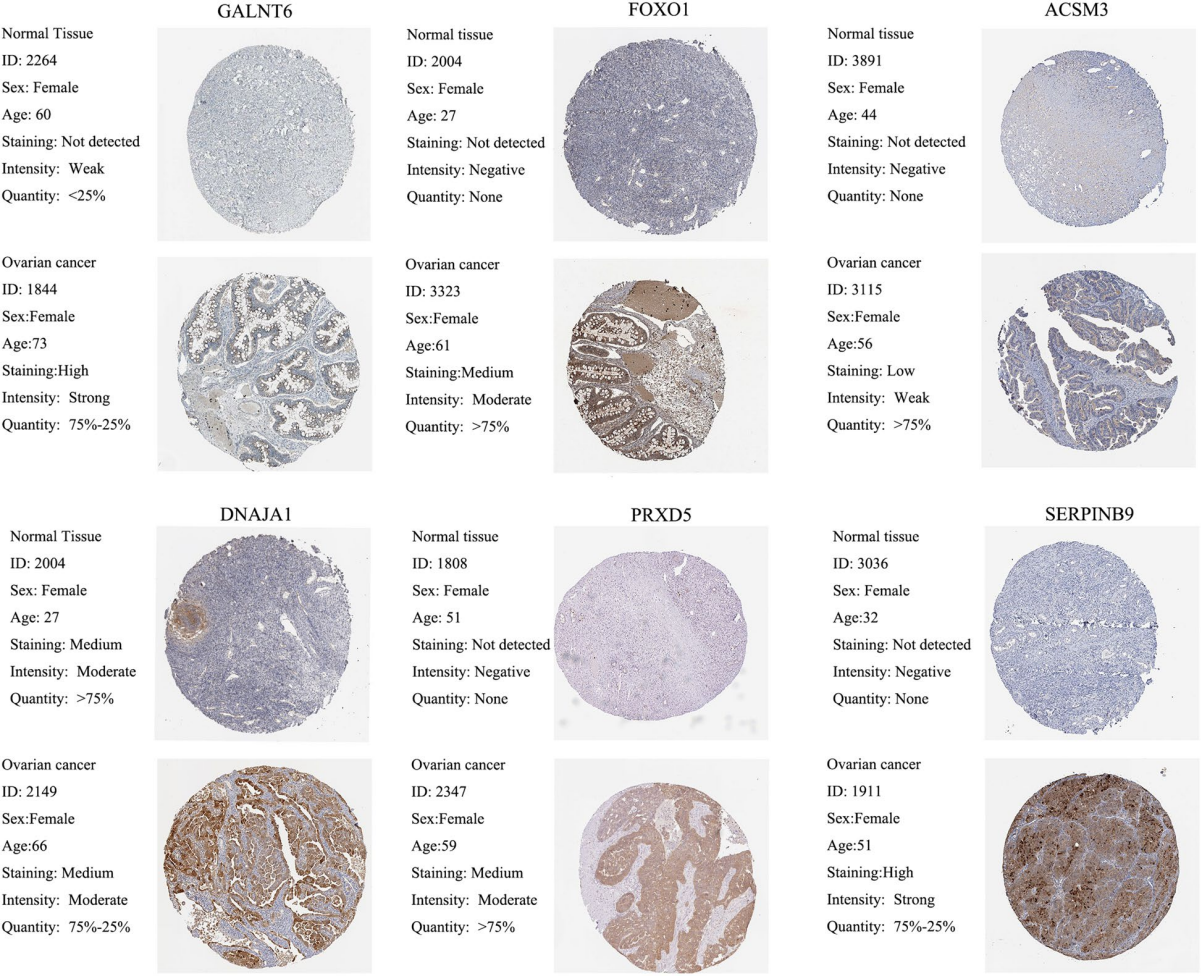
Protein expression of 10 crucial genes in epithelial ovarian carcinoma and normal ovary tissues based on HPA database

We examined the protein expression of 10 genes in normal and tumour tissues and presented an immunohistochemical graph from the HPA database shown in Fig. 8. Compared with normal tissues, six proteins (GALNT6A, FOXO1, ACSM3, DNAJA1, PRDX5, and SERPINB9) were notably overexpressed in tumour tissues. Immunohistochemical images of the remaining four genes are shown in Supplementary Fig. 2. The elevated expression of these genes was related to poor prognosis, which is compatible with our results.

## Discussion

Epithelial ovarian carcinoma has the highest mortality rate among gynaecological malignancies [40]. An effective and sensitive diagnosis remains challenging in the early stages, and chemotherapy resistance, along with metastasis, contributes to poor therapeutic efficacy and prognosis in advanced stages [41]. Therefore, a rapid and accurate early diagnosis, coupled with the implementation of rational drug therapy strategies, is crucial for the treatment of EOC. Epigenetics is essential for tumourigenesis and progression, with numerous genes associated with epigenetic modifications frequently altered in cancer. These genes may act as driver genes in oncogenic process [42]. Currently, there is increasing interest in investigating the interplay between acetylation and its effects on tumour development, metastasis, and progression [43], including in EOC [44]. Therefore, targeting the genes involved in acetylation is a promising strategy for future treatment.

Over the past decade, ICB immunotherapies targeting PD-1 and PD-L1 have rapidly evolved for use with EOC patients. Clinical trials on Olaparib, Rucaparib, and Niraparib [45] have yielded encouraging results. Immunotherapy is emerging as a prominent and novel treatment option for patients with high-grade metastatic disease. Owing to the molecular heterogeneity of EOC, only approximately 20% of patients exhibited a positive response to ICB therapy. Therefore, identifying genomically characterised biomarkers is imperative for accurate prediction and selection of patients likely to achieve favourable outcomes with immunotherapy. Similarly, certain biomarkers can help stratify patients and predict their responses to chemotherapy. For instance, the presence of either BRCA gene mutations or homologous recombination deficiency (HRD) is a widely used biomarker for poly (ADP-ribose) polymerase (PARP) inhibitors, with BRCA gene mutations being the preferred sensitive biomarkers for PARP inhibitor therapy [46]. Consequently, studies focused on identification of effective biomarkers of chemotherapy and immunotherapy are urgently needed.

Though previous studies have explored the crucial role of acetylation in EOC, most focused on a restricted number of acetylation-associated proteins. The complete function of acetylation in EOC and the interaction between acetylation and TME cell infiltration have not yet been elucidated. Therefore, there is a need for more effective immunotherapy, chemotherapy, and treatments targeting acetylation.

In this study, we explored the differences in the activation pathways of differentially expressed acetylation-related genes associated with prognosis. The genes were categorised into three acetylation patterns, termed as acetylation C1, C2, and C3. Ten genetic markers were constructed using Lasso-Cox regression modelling and were demonstrated to be independent risk factors for EOC. Based on the risk scores generated by the model, patients could be separated into two subgroups: high- and low-risk. The OS of patients exhibited significant differences in the two subgroups. C1 and C2 demonstrated a noticeable enrichment in immune-related pathways based on the KEGG analysis. C3 was more active in tumour cell proliferation-related pathways, and these differences were statistically significant. The activation of the Hedgehog signalling pathway was found to be significantly increased in the C3 pattern. Prior studies have demonstrated that the Hedgehog signalling pathway is aberrantly activated in EOC, increases the incidence of EOC, and leads to a poorer prognosis [47]. Conversely, the precise function of Hh signalling in the development and prognosis of EOC remains unclear. Despite the absence of obvious differences in clinical characteristics, significant differences in TME immune cell infiltration and overall survival were observed among the three acetylation-related clusters and the two risk groups. T cells, follicular helper cells, activated dendritic cells, and M1 macrophages exhibited high levels of infiltration in the low-risk group. C2 presented with a poorer survival prognosis even though it was enriched with abundant immune cells. TAMs and Tregs may play key roles in promoting the malignant progression of EOC. TAMs and Tregs are important immunosuppressive cells that may lead to tumour metastasis, drug resistance, angiogenesis, and immune escape, resulting in poor prognosis [48–50]. Many studies have shown that acetylation is important for the function and stability of TAMs and Tregs. Moreover, BBR inhibits inflammatory response in TAMs. This decreases the acetylation of p65^Lys310^ by downregulating the activity of p300. Furthermore, it inhibits the transcriptional activity and translocation of NF-κB [51]. It was also found that NAC1 reduced the stability mediated by acetylation of the housekeeping protein FoxP3 in Treg cells, inhibiting Treg cell development and leading to tumourigenesis [52]. Therefore, we suggested that the acetylation pattern exhibited a strong correlation with the infiltration of immune cell into the TME.

Many patients benefit from immunotherapy using ICB (e.g. PD-1, PD-L1, and CTLA-4 blockade), whereas others do not demonstrate a significant clinical response to this treatment. PD-1/PD-L1 blockade has shown marked efficacy as a second-line therapy, whether as monotherapy or in combination with chemotherapy or CTLA-4 checkpoint inhibition, in patients with EOC. In the cisplatin-resistant group, a phase II clinical trial employing the PD-1 inhibitor pembrolizumab plus niraparib showed an objective remission rate (ORR) of 21% [53, 54]. In another phase II clinical trial, the combination of olaparib with the PD-L1 inhibitor durvalumab showed an ORR of 15% [55, 56]. These results suggest that remission rates remain low, emphasizing the need to screen patients suitable for immunotherapy. Our results showed that C1 was associated with an enhanced response to PD-1 blocker immunotherapy. Data from the IMVigor 210 cohort was employed to validate the immunotherapy responses in all three groups, consistent with previous test results. Additionally, the immunoinflammatory phenotype was significantly enriched in C1, indicating a greater reactivity to immunotherapy. Consequently, we believe that acetylation models play a significant role in distinguishing various TIME and may serve as a reliable biomarker for selecting patients suitable for immunotherapy. In addition, TMB serves as a potent biomarker for forecasting the efficacy of immune checkpoint inhibitor treatment in EOC patients [57]. We further explored the relationship of TMB between the three clusters and the two risk groups, considering the complexity of acetylation and individual heterogeneity. The TMB of C1 tumours and the low-risk group exhibited a notable increase, suggesting that acetylation patterns accurately characterise TME cell infiltration in individual patients.

Meanwhile, we also investigated the correlation between acetylation and the chemotherapeutic response. Cisplatin, paclitaxel, gemcitabine, capecitabine, etoposide, bevacizumab, and oxaliplatin are commonly administered as conventional chemotherapies for EOC. These are gradually being replaced by newer regimens, including cisplatin plus paclitaxel and paclitaxel in combination with bevacizumab. We assessed the correlation between acetylation and sensitivity to chemotherapeutic agents. The IC_50_ values of all these drugs exhibited apparent differences among the three clusters, suggesting that acetylation patterns play a unique role in predicting chemotherapeutic efficacy and guiding clinical treatment.

Among the polygenic markers we established, four risk factors ( FOXO1, KYAT1, JUP, and DYRK1B) and six protective factors ( GALNT6, ACSM3, DNAJA1, PRDX5, TPMT, and SERPINB9) were identified. In previous studies, the enzyme N-Acetylgalactosaminyltransferase 6 (GALNT6) has been shown to have a crucial role in the initial step of mucin-type O-glycosylation. Its involvement has been linked to the recurrence, lymph node metastasis, and chemoresistance of EOC by modulating EGFR activity, resulting in poor prognosis [58]. The transcription factor forkhead box protein O1 (FOXO1) functions as a transcriptional repressor of T-cell activation program [59] and serves as a suppressor of natural killer cell maturation and function [60]. It promotes the differentiation of regulatory T and B cells, inhibits the formation of T helper cells 1 (Th1) and Th17 cells, and is activated by dendritic cells (DC). Studies have shown that FOXO1 plays a key role as a downstream factor of the PI3K/Akt pathway in EOC. GnRH agonists may promote apoptosis in EOC cells through the upregulation of FOXO1 in the PI3K/AKT signalling pathway, making FOXO1 a promising target for therapeutic intervention in the management of EOC [61]. Low FOXO1 expression was observed in platinum-resistant EOC, suggesting that FOXO1 could be targeted for earlier diagnosis and more accurate treatment of EOC [62]. However, FOXO1 was identified as a risk factor in this work. Previous studies have indicated that FOXO1 inhibits the progression of EOC, which may cause worse prognosis. However, the precise function of FOXO1 in the progression and outcome of EOC remains unclear. The tumour suppressor gene ACSM3 can inhibit the proliferation, migration, and invasion of EOC cells and may be a therapeutic target for EOC [63]. The expression of PRDXs family proteins is increased in ovarian cancer tissues in comparison to normal ovarian tissues. Furthermore, heightened expression of PRDX5 leads to unfavourable progression-free survival (PFS) outcomes in EOC patients [64]. DYRK1B is usually amplified or upregulated in EOC, making it a potential therapeutic target for EOC ascites [65]. Other researchers have demonstrated the utility of SERPINB9 immunotherapy as a novel candidate to modulate ICB resistance [30]. The results of analysis of these genes broadly match the results of this work.

We explored the interaction between the risk group and clinical features, finding significant correlations of acetylation patterns with age, state, grade, and risk score. Survival analysis revealed that the two risk groups were excellent predictors of the survival index. A nomogram model was developed using four key characteristics (age, grade, stage, and risk score), enabling the prediction of the likelihood of survival for patients with EOC at 1, 5, and 10 years. The ROC correction curve and AUC showed robust discriminant abilities.

## Conclusions

Our study provides profound insights into the interactions involving acetylation, TME, TMB, and responses to chemotherapy and immunotherapy. We demonstrated the utility of acetylation-related genes in distinguishing patterns of immune cell infiltration in TME and clinical features, as well as their predictive capabilities for responses to ICBs and chemotherapy. These studies contribute to enhancing clinical treatment strategies and patients screening for immunotherapy or chemotherapy, guiding future precision therapies.

Nevertheless, we acknowledge limitations in our research. Recent discoveries of acetylation-related proteins imply that our collected genes may not be comprehensive enough, potentially introducing biases. The limited number of clinical samples and the absence of validation in other clinical databases, aside from public data, are additional constraints. To address these limitations, we intend to collect more clinical samples for validation and extend our investigation to other reproductive tumours. Finally, the detailed mechanism by which acetylation patterns interact with TMB immune cell penetration is not fully understood, warranting further research.

### Supplementary Information

Supplementary Material 1. Supplementary Figure 1. The diagram of the full workflow. 8+ Supplementary Figure 2. Differentially expressed proteins of crucial genes in ovary carcinoma and normal ovary tissues in the Human Protein Atlas database. Supplementary Figure 3. Correlation between differential genes. GALNT6A, FOXO1, ACSM3, DNAJA1, KYAT1, JUP, PRDX5, TPMT, DYRK1B, and SERPINB9 are genes with low covariance and are independent of each other

Supplementary Material 2.

## Data Availability

No datasets were generated or analysed during the current study.
